# MeatScan: An image dataset for machine learning-based classification of fresh and spoiled cow meat

**DOI:** 10.1016/j.dib.2025.112045

**Published:** 2025-09-08

**Authors:** Rose-Mary Owusuaa Mensah Gyening, Michael Appiah Akoto, Kwabena Owusu-Agyemang, Linda Amoako-Banning, Kate Takyi, Peter Appiahene

**Affiliations:** a Kwame Nkrumah University of Science and Technology, Department of Computer Science, Ghana; b University of Energy and Natural Resources, Department of Computer Science and Informatics, Ghana

**Keywords:** Deep learning, Image classification, Cow meat spoilage, Food quality assessment, Convolutional neural networks, Machine learning

## Abstract

This article presents MeatScan**,** a curated image dataset developed to support deep learning-based binary classification of cow meat as fresh or spoiled. The dataset comprises 11,000 high-resolution RGB images (5627 fresh and 5373 spoiled) captured in real-world Ghanaian environments, including open-air markets, butcher shops, and cold storage facilities. Images were labeled based on observable visual cues such as texture, colour, and surface condition, with annotations verified under natural lighting by trained data collectors. MeatScan provides structured and contextually rich visual data for supervised learning in food quality monitoring. It addresses a key gap between advances in computer vision and practical food safety inspection, especially in low-resource settings. The dataset supports experimentation with convolutional neural networks, transfer learning, and data augmentation, and serves as a real-world benchmark for evaluating model robustness to lighting variability, diverse meat textures, and complex backgrounds.

Specifications Table

**Table:** 

Subject	Computer Sciences
Specific subject area	Image Classification of Meat Spoilage
Type of data	Image, Metadata (CSV)
Data collection	Images were captured in Kumasi, Ghana, using Tecno Camon 15 (48 MP) and Infinix Smart 5 (13 MP) smartphone cameras under natural daylight or ambient indoor lighting. Only clear images of raw cow meat labeled fresh or spoiled were included; blurred or obscured images were excluded.
Data source location	• Institution: Kwame Nkrumah University of Science and Technology • City/Town/Region: Ashanti Region • Country: Ghana
Data accessibility	Repository name: Zenodo Data identification number: https://doi.org/10.5281/zenodo.16764338 Direct URL to data: https://doi.org/10.5281/zenodo.16764338
Related research article	None

## 1 Value of the Data

• This dataset supports the development and evaluation of deep learning models for binary classification of fresh and spoiled cow meat
• It facilitates rapid, objective, and scalable meat spoilage detection, overcoming manual inspection methods that are subjective and inconsistent.
• It enables practical AI research in food safety inspection, especially in low-resource environments lacking laboratory infrastructure.
• It can be used to train and benchmark CNNs, transfer learning models, and mobile-ready lightweight classifiers.
• Researchers can use this dataset to test model robustness against lighting variations, meat texture inconsistencies, and complex backgrounds.
• It serves as a real-world reference dataset for computer vision applications in food safety, agriculture, and public health.

## 2 Background

Deep learning is a powerful approach for food safety automation, but is seldom used in practice due to a lack of contextually diverse, domain-specific datasets. The majority of currently available meat spoilage datasets are limited in size, created in controlled environments, or lack annotations that correspond to spoilage variation in informal markets. For instance, the Meat Quality Assessment Dataset by [1] contains supermarket-acquired images under controlled lighting, while the IoT-Based Meat Freshness Dataset by [2] combines visual and sensor data (e.g., gas sensors) to assess spoilage. Other datasets, such as those using hyperspectral imaging, are valuable for scientific modeling but require specialized equipment not suited for deployment in resource-limited settings [3,4]. Despite these contributions, most existing datasets are constrained by either their artificial environments, limited regional diversity, or reliance on technologies that are impractical in low-income markets. Machine learning-based visual inspection is faster, more objective, scalable, and can operate more adequately in low-resource settings than traditional visual inspection. This offers a practical pathway for consistent and reliable meat quality evaluation, allowing for automated meat classification.

The pre-existing datasets do not adequately capture the contextual realities of cow meat spoilage in West African markets. Most existing datasets were acquired under highly controlled laboratory conditions or rely heavily on gas sensors and spectroscopy equipment, which are impractical for rapid, scalable deployment in informal or low-resource settings. Furthermore, publicly accessible datasets containing high-resolution, visually distinguishable images of fresh and contaminated cow meat from the African region are virtually non-existent. Also, according to [5], meat handling practices in Africa pose systemic risks to public health, calling for artificial intelligence-enabled inspection systems. Similarly, [6] emphasized that Uganda’s meat supply chain lacks technological infrastructure. MeatScan contributes toward addressing this gap by introducing a large-scale, publicly accessible dataset of fresh and spoiled cow meat images, collected from natural Ghanaian market settings using smartphones.

In Ghana and across sub-Saharan Africa, beef remains a major component of daily nutrition and a key commodity in informal markets. Yet, challenges such as poor meat handling, limited refrigeration, and weak inspection protocols allow spoiled meat to circulate freely, increasing public health risks [5,6]. Visual inspection remains the primary method of assessment in these contexts, yet it is subjective and unreliable under inconsistent lighting and handling conditions. Existing image datasets are frequently restricted to fruits and vegetables or are gathered in controlled settings, which means they do not accurately represent real-world market conditions, even with advances in deep learning for food quality assessment [7,8].

To bridge this gap, this study presents MeatScan, a curated dataset of 11,000 high-resolution RGB images of fresh and spoiled cow meat captured in real-world Ghanaian markets, cold rooms, and butcher shops. MeatScan was built using low-cost smartphones in uncontrolled environments, preserving the visual variability seen in day-to-day market settings. It supports binary classification tasks (fresh vs. spoiled), encourages the development of AI-driven meat inspection tools, and reflects the practical realities of informal food systems in low-resource settings. A comparative overview of related meat inspection datasets is summarized in Table 1.

**Table 1:** Overview of related meat inspection datasets.

Dataset Name	No. of Images	Resolution	Labels	Modalities
Meat Quality Assessment [1]	1896	1280×720	Fresh/Spoiled	RGB
Meat Freshness Image [9]	2266	416×416	Fresh/Half-Fresh/Spoiled	RGB
IoT-Based Freshness [2]	6672	Varying	Fresh/Spoiled+ Species	RGB + Gas Sensors
Beef Spoilage HSI [4]	5867	640×640	Fresh / Spoiled	RGB + Augmentations

MeatScan was curated to facilitate deep learning-based research on meat spoilage image classification. MeatScan offers a dataset of raw cow meat samples taken in realistic, everyday settings, in contrast to many other datasets photographed in sterile, lab-controlled settings. With its detailed annotations and high-resolution imagery, the dataset serves as a valuable resource for stakeholders spanning public health, food technology, computer vision, and artificial intelligence research. It can facilitate the development of practical commercial applications, including AI-powered market monitoring systems, embedded inspection cameras, and mobile-based spoilage detection tools, as well as scholarly investigation of model architectures. The dataset also advances technological inclusion by promoting the adoption of AI within conventional food supply systems, particularly in contexts historically underserved by such innovations. This facilitates data-driven decision-making across traditionally manual stages of the food supply chain.

## 3 Data Description

The MeatScan dataset was curated using images collected from diverse environments that reflect the real-world conditions in which cow meat is sold and handled in Ghana. Data collection took place across multiple open-air markets, cold rooms, and butcher shops. The goal was to ensure the dataset captures a wide range of environmental variations such as lighting differences, surface backgrounds, and moisture conditions, which are crucial for training robust computer vision models. Image acquisition was conducted by trained personnel who photographed both fresh and spoiled meat samples at different times of the day to maximize lighting variability. At the point of capture, the collected images were labelled based on visible spoilage characteristics such as discoloration, sliminess, mold, and dryness, as informed by field experts. This approach ensured that image annotations were grounded in practical domain knowledge relevant to meat hygiene.

The novel dataset created for this study consists of 11,000 RGB images of raw cow meat, categorized into two distinct classes: Fresh and Spoiled. A total of 3487 images belong to the Fresh class, while 3508 are labelled as Spoiled. The dataset includes a total of 4005 augmented images, comprising 2140 fresh and 1865 spoiled samples generated through flipping, rotation, and brightness alteration. These augmentations were leveraged to increase the diversity of the dataset and the generalization ability of machine learning models. The augmented images are uniformly divided into the Fresh (2140 images) and Spoiled (1865 images) classes as shown in Table 2. This near-balanced distribution reflects dataset design aimed at supporting stable binary classification. The relatively equal representation of both classes enhances model training dynamics by minimizing the risk of class bias, thereby improving generalization and predictive reliability across real world contamination scenarios.

**Table 2:** Folder structure of the MeatScan dataset showing both original and synthetically augmented images.

SN	Name	Folder Name	Original images	Additional Images from Data Augmentation
1	Fresh	Fresh_CowMeat	3487	2140
2	Spoiled	Spoiled_CowMeat	3508	1865
**Total**			**6995**	**4005**

**Fig. 1:**
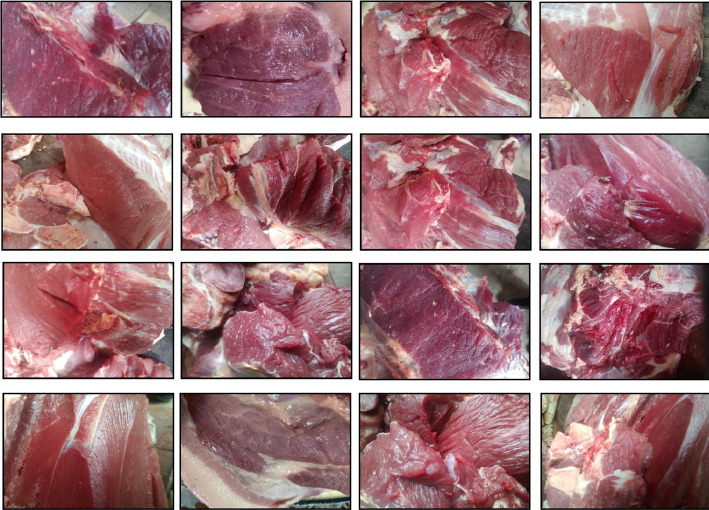
Sample images of the fresh cow meat class.

**Fig. 2:**
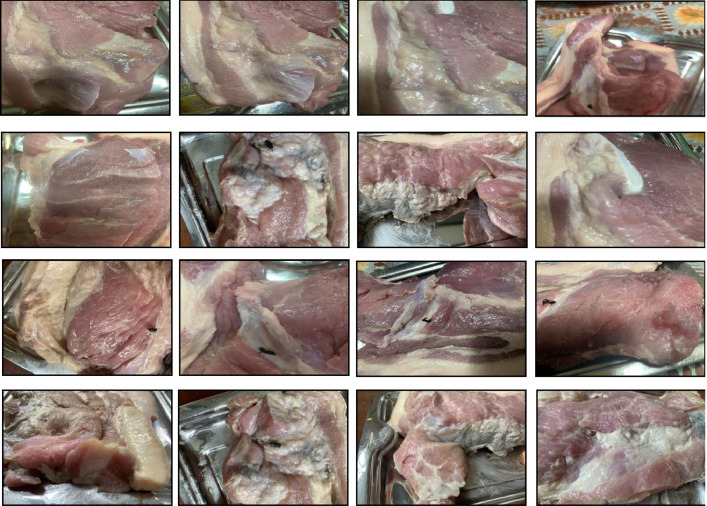
Sample images of the spoiled cow meat class.

Uniformity in image dimensions was ensured by resizing all images to 224 × 224 pixels. The dataset includes significant intra-class variation across both categories. These variations span lighting conditions, meat surface textures, camera angles, and sample arrangements. Such variations were deliberately introduced to improve model robustness under non-standardized field conditions. Fig. 1, Fig. 2 present a visual overview of representative samples from both fresh and spoiled cow meat classes, respectively.

**Fig. 3:**
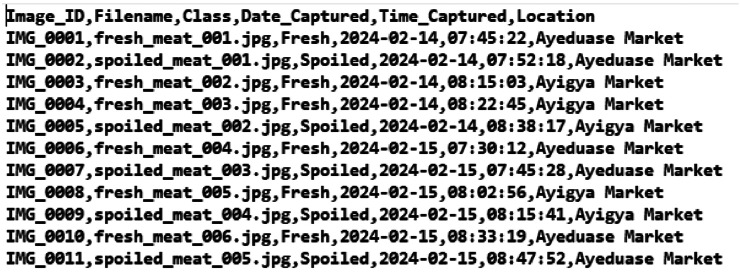
Snapshot of the CSV metadata of MeatScan dataset.

All image samples were systematically stored in class-specific directories, namely, Fresh and Spoiled, to ensure proper dataset organization and efficient access during model training. In addition to image storage, a metadata file in CSV format was maintained to record contextual information associated with each image. This metadata includes the image’s date of capture, time of capture, and the geographic location where it was acquired. Each image is uniquely identified and tagged with these attributes, supporting chronological sorting, traceability, and reproducibility during experimentation. Fig. 3 presents a snapshot of the CSV metadata of the dataset and Table 2 presents the folder structure of the MeatScan dataset.

High data quality is essential for both model training and evaluation. Stringent quality control measures began with an initial image screening process, where low-resolution, blurry, or noise-distorted images were systematically excluded to ensure high visual fidelity. Duplicate detection techniques were also applied to identify and remove identical images, reducing data leakage and class imbalances. Consistency in labeling was validated through inter-rater agreement analysis involving three independent annotators. To reduce bias, a washout period of two weeks was provided after a random subset of 1200 class images, (600 images each from the fresh and spoiled classes) were assessed by annotators. The agreement among the three annotators was evaluated using Fleiss’ Kappa. The resulting score of κ = 0.87 demonstrates strong agreement beyond chance.

The high Fleiss’ Kappa score demonstrates that the annotators consistently applied similar labeling criteria when classifying images, indicating that the class labels in the MeatScan dataset can be considered accurate and reliable. Moreover, the category-specific criteria for labeling decision-making regarding spoilage were mainly based on visual cues such as discoloration, texture degradation, and dryness, which are also perceived as visual hygiene parameters by field inspectors in local markets. This repeated review process improved label reliability by reducing the likelihood of annotation errors or inconsistencies. The consistent application of these quality control steps ultimately contributes to the robustness of MeatScan and enhances the reliability of models trained on it.

The MeatScan dataset offers substantial value to researchers and practitioners at the intersection of computer vision, food safety, and public health. It is designed primarily to support the training and evaluation of deep convolutional neural networks (CNNs) on binary classification tasks, specifically for distinguishing between fresh and spoiled cow meat. The dataset comprises a cleanly labeled and diverse collection of real world images, enabling rigorous assessment of how different CNN architectures perform on food quality classification tasks. Its binary labeling structure facilitates precise model evaluation and fine-tuning, especially for safety-critical applications where accurate decisions are essential. The images were captured under varying lighting and environmental conditions, ranging from indoor cold rooms to naturally lit open markets and dimly lit butcher shops. These variations introduce real world noise, enhancing the dataset’s capacity to support robust model generalization across diverse operational domains. This is particularly important for deployment in uncontrolled settings, such as rural or informal food distribution chains.

Advanced machine learning approaches, including transfer learning, data augmentation, and attention-based mechanisms, can benefit significantly from large, high-resolution, and context-rich datasets like *MeatScan*. The dataset enables exploration of fine-grained visual features related to meat spoilage and freshness. It is especially suited for the development of lightweight models optimized for mobile deployment, supporting real time meat quality detection on low-cost smartphones or embedded systems.

This dataset addresses a major challenge in low-resource settings, where formal meat inspection infrastructure is often lacking or inconsistently applied. *MeatScan* lays the groundwork for the development of intelligent, affordable, and portable food safety tools, helping bridge the technological divide. Its application can contribute to measurable improvements in meat handling practices, market transparency, and consumer health protection. Finally, *MeatScan* serves as a standardized reference for computer vision research in food technology and agriculture.

In the emerging field of AI-enabled food inspection, it promotes reproducible research, supports comprehensive experimentation, and provides a reliable benchmark for developing real time meat spoilage detection systems. Beyond binary classification, the MeatScan dataset can also serve as a foundation for a range of machine learning tasks. These include feature extraction and representation learning for detecting spoilage patterns, domain adaptation where models trained on controlled datasets are fine-tuned for real-world deployment, and explainability research that aims to visualize and interpret model decisions in food safety contexts.

Additionally, the dataset is well-suited for training and evaluating lightweight neural networks for edge deployment, image segmentation models to localize spoilage regions, and multi-class extensions that may incorporate intermediate freshness levels in future versions. Researchers can also use MeatScan to explore robustness testing under various augmentations, conduct cross-dataset generalization experiments, and benchmark novel few-shot learning or semi-supervised learning approaches, particularly when working with limited labeled samples.

## 4 Experimental Design, Materials and Methods

In an effort to mirror data conditions prevalent in real-world, low-resource environments, images were captured using widely available smartphones, including the Tecno Camon 15, Infinix Smart 5, LG V50S ThinQ, and Samsung S21, with camera resolutions between 8MP and 13MP. These devices were chosen to reflect the average consumer-grade imaging tools accessible in local markets. To maintain the authenticity of the environmental conditions, only ambient light, natural sunlight, or indoor fluorescent lighting was used during the image collection phase. Neither external lighting nor flash systems were used.

**Fig. 4:**
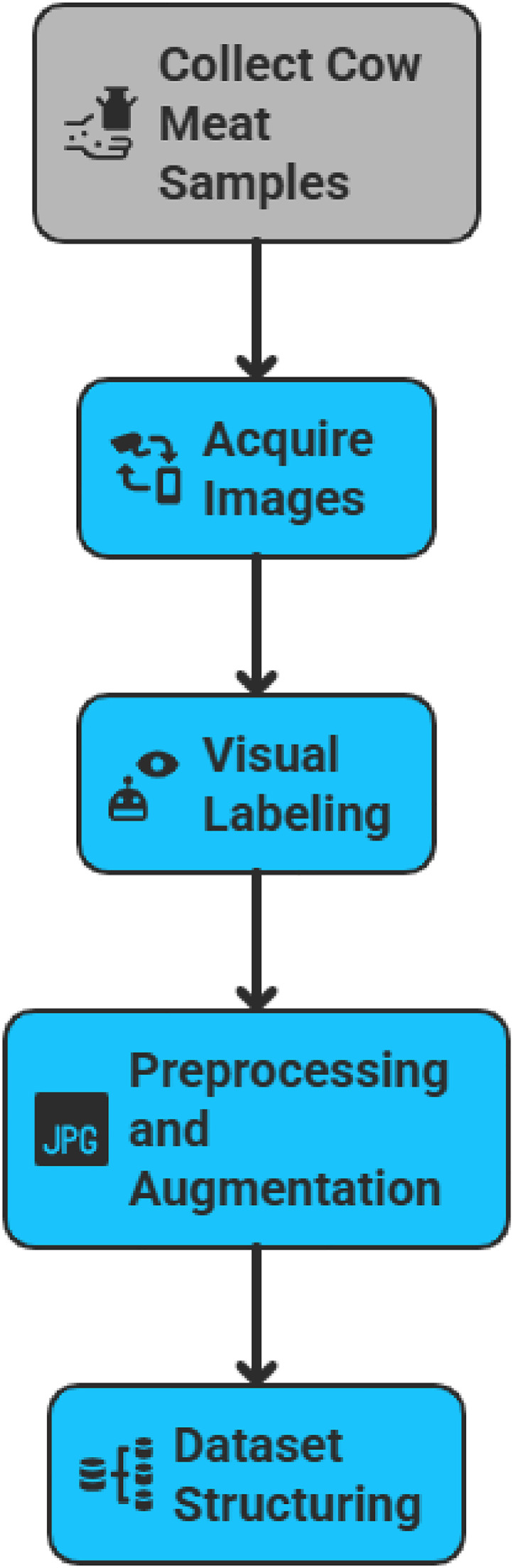
Workflow for the MeatScan image dataset generation.

This setup aligns with the broader objective of deploying deep learning-based meat freshness classifiers on embedded and mobile systems, particularly in contexts where advanced imaging infrastructure is unavailable. Using commonly accessible devices ensures that the trained models remain compatible with images captured by everyday users, eliminating the need for specialized equipment. The full specifications of the camera setup used in this study are detailed in Table 3. Fig. 4 illustrates a conceptual workflow outlining the image acquisition process for the MeatScan dataset.

**Table 3:** Smartphone camera specification used for MeatScan image acquisition.

Attribute	Specification
Devices Used	Tecno Camon 15, Infinix Smart 5, LG V50S ThinQ, Samsung S21
Camera Resolution	13MP (rear) and 8MP (front)
Sensor Type	CMOS (consumer-grade)
Auxiliary Light Source	None (ambient light only; natural or fluorescent)
Digital Zoom	Up to 4× (used sparingly; focus on native resolution)
Image Format	JPEG (.jpg)
Image Resolution (saved)	1920 × 1080 (scaled later to 224 × 224)
Stabilization	Handheld, no tripod or stabilization accessories
Interface	Android OS, internal storage, USB 2.0 for transfer
Capture Method	Manual, using stock camera app

To demonstrate that the MeatScan dataset can be effectively used for binary classification tasks, a baseline experiment was conducted using MobileNetV2. A replicated experimental design with three independent training runs was implemented to account for variability in training. MobileNetV2 was selected because of its lightweight architecture and suitability for mobile deployment in low-resource settings. The model was fine-tuned using transfer learning with input images resized to 224×224 pixels. The dataset was split into 80 % for training and 20 % for validation to ensure balanced representation and minimization of sampling bias

The model was trained using the Adam optimizer, with a learning rate of 0.0001, batch size of 32, and binary cross-entropy loss function over 30 epochs. To enhance generalizability and reduce overfitting, data augmentation techniques such as random flipping, brightness enhancement, and image rotation were applied during training. Classification accuracy was the primary evaluation metric, while precision, recall, and F1-score were considered additional metrics. Across the three experimental replicates, the MobileNetV2 model achieved an average accuracy of 92.3 % with a precision of 91.5 %, a recall of 92.9 % and an F1-score of 92.2 %.

The MeatScan dataset is a high-quality open-access dataset that contributes to addressing the gap between academic research and practice in AI-driven food safety. It is reliable for image-based meat spoilage classification. It could also serve as a benchmark dataset for evaluating the performance and robustness of alternative models or deployment-ready AI solutions in food safety applications.

## Limitations

The MeatScan dataset makes a significant contribution to the field of food quality classification; however, several limitations are acknowledged. The dataset is currently limited to Ghana, which may restrict its generalizability to regions with different climatic or spoilage conditions. Spoilage annotations are based on visual inspection, introducing the potential for minor inconsistencies or subjectivity. At present, MeatScan supports only binary labels, namely fresh and spoiled, without capturing finer-grained freshness categories. It also lacks environmental metadata such as temperature, humidity, and time-based indicators of spoilage progression. Additionally, expanding the dataset to include more meat types, varied camera angles, and surface backgrounds could enhance its robustness and applicability in future versions.

## Ethics Statement

The authors confirm that they have read and comply with the ethical requirements for publication in Data in Brief. This work does not involve human subjects, animal experiments, or any data collected from social media platforms.

## CRediT Author Statement

**Rose-Mary Owusuaa Mensah Gyening:** Conceptualization, Methodology, Data curation, Metadata preparation, Writing – original draft, Writing – review & editing, Supervision. **Michael Akoto Appiah:** Data collection, Annotation, Validation, Data curation, Writing – original draft, Writing – review & editing. **Kwabena Owusu-Agyemang:** Data collection, Annotation, Writing – original draft, Writing – review & editing. **Linda Amoako-Banning:** Data collection, Annotation, Writing – original draft, Writing – review & editing. **Kate Takyi:** Data collection, Annotation, Writing – original draft, Writing – review & editing. **Peter Appiahene:** Data collection, Annotation, Writing – original draft, Writing – review & editing.

## Data Availability

ZenodoMeatScan: An Image Dataset for Machine Learning-Based Classification of Fresh and Spoiled Cow Meat (Original data).
